# Modified connectivity of vulnerable brain nodes in multiple sclerosis, their impact on cognition and their discriminative value

**DOI:** 10.1038/s41598-019-56806-z

**Published:** 2019-12-27

**Authors:** Elisabeth Solana, Eloy Martinez-Heras, Jordi Casas-Roma, Laura Calvet, Elisabet Lopez-Soley, Maria Sepulveda, Nuria Sola-Valls, Carmen Montejo, Yolanda Blanco, Irene Pulido-Valdeolivas, Magi Andorra, Albert Saiz, Ferran Prados, Sara Llufriu

**Affiliations:** 1 Center of Neuroimmunology, Laboratory of Advanced Imaging in Neuroimmunological Diseases, Hospital Clinic Barcelona, Institut d’Investigacions Biomèdiques August Pi i Sunyer (IDIBAPS) and Universitat de Barcelona, Barcelona, Spain; 2 E-health Centre, Universitat Oberta de Catalunya, Barcelona, Spain; 3 Centre for Medical Image Computing (CMIC), Department of Medical Physics and Bioengineering, University College London, London, UK; 4 NMR Research Unit, Queen Square MS Centre, Department of Neuroinflammation, UCL Institute of Neurology, University College London, London, UK

**Keywords:** Cognitive neuroscience, Diffusion tensor imaging, Multiple sclerosis, Machine learning

## Abstract

Brain structural network modifications in multiple sclerosis (MS) seem to be clinically relevant. The discriminative ability of those changes to identify MS patients or their cognitive status remains unknown. Therefore, this study aimed to investigate connectivity changes in MS patients related to their cognitive status, and to define an automatic classification method to classify subjects as patients and healthy volunteers (HV) or as cognitively preserved (CP) and impaired (CI) patients. We analysed structural brain connectivity in 45 HV and 188 MS patients (104 CP and 84 CI). A support vector machine with *k*-fold cross-validation was built using the graph metrics features that best differentiate the groups (p < 0.05). Local efficiency (LE) and node strength (NS) network properties showed the largest differences: 100% and 69.7% of nodes had reduced LE and NS in CP patients compared to HV. Moreover, 55.3% and 57.9% of nodes had decreased LE and NS in CI compared to CP patients, in associative multimodal areas. The classification method achieved an accuracy of 74.8–77.2% to differentiate patients from HV, and 59.9–60.8% to discriminate CI from CP patients. Structural network integrity is widely reduced and worsens as cognitive function declines. Central network properties of vulnerable nodes can be useful to classify MS patients.

## Introduction

Multiple sclerosis (MS) is a chronic inflammatory and neurodegenerative disease of the central nervous system that leads to physical and cognitive disability. Using diffusion-magnetic resonance imaging (MRI), abnormalities in structural brain connectivity have been seen to be driven by demyelinating and neuroaxonal damage in patients with MS^1–4^. Likewise, graph theory analyses of the structural brain network suggests that MS is associated with an imbalance in the integration and segregation of the network components^3^, which deteriorates the information flow among brain regions^1,2^, and affects their function^3,4^. In this sense, cognitive impairment has been related to a decrease in network efficiency, and changes in nodes and connections involving the insula, deep grey matter and regions of the frontoparietal network^1,2^. Measures of small-worldness and network segregation, such as local efficiency or clustering coefficient, can provide regional information by characterising the interactions of an individual node with its immediate neighbours^5^. Such network parameters may be modified by the disease and lead to cognitive dysfunction when the network collapses^6^.

Using machine learning algorithms, network characteristics have been used in preliminary studies to discriminate between patients and healthy subjects^7–9^, to depict disease evolution^8^, or to classify in the different clinical profiles of MS^7^. Among other classification techniques, Support Vector Machines (SVM^10^) are efficient, robust and accurate methods to classify data in diverse and heterogeneous contexts, such as those related to medical diagnosis^11,12^. Thus, the application of such methods may aid MS diagnosis and enhance our understanding of the network components that collapse when cognitive dysfunction is associated with the development of this disease. Therefore, the aim of this study was to characterise the regional changes in connectivity in patients with MS that influence their cognitive status. As such, using a supervised learning model we set out to provide an automatic classification method based on this information that is capable of distinguishing MS patients from healthy individuals, and of distinguishing cognitively impaired (CI) and cognitively preserved (CP) MS patients.

## Methods

### Participants

Patients with relapsing-remitting or secondary progressive MS, aged between 18 and 65 years, were recruited to this study at the MS Unit of the Hospital Clinic of Barcelona. We included 188 MS patients diagnosed according to the 2010 McDonald criteria^21^ and 45 HV with no previous or current history of neurological or psychiatric diseases. Physical disability was evaluated in all the subjects using the Expanded Disability Status Scale (EDSS). The Ethics Committee of the Hospital Clinic of Barcelona approved the study, and all participants signed an informed consent. All procedures of this study were performed in accordance with the relevant guidelines and regulations.

### Cognitive assessment

The Brief Repeatable Battery of Neuropsychological tests (BRB-N^22^) was completed by all patients, and the z-scores were calculated using age and education-adjusted norms^23^. Patients were classified as CI when they had a z-score below −1.5 in two or more tests, while the remaining patients were classified as CP.

### Magnetic resonance image acquisition and processing

#### Structural and diffusion-magnetic resonance image acquisition

MRIs were acquired on a 3 T Magnetom Trio scanner (SIEMENS, Erlanger, Germany) using a 32 channel phased-array head coil as previously described^2,16^; including structural 3D-Magnetization Prepared Rapid Acquisition Gradient Echo (MPRAGE), 3D-T2 fluid-attenuated inversion recovery (FLAIR) and diffusion-weighted imaging (DWI) sequences. The 3D-structural image had the following acquisition parameters: TR = 1800 ms; TE = 3.01 ms; TI = 900 ms; 240 sagittal slices with 0.94 mm isotropic voxel size and a 256 × 256 matrix size. The 3D-T2 FLAIR sequence parameters were: TR = 5000 ms; TE = 304 ms; TI = 1800 ms; 192 sagittal slices with 0.94 mm isotropic voxel size and a 256 × 256 matrix size. DWI was acquired with TR = 14800 ms; TE = 103 ms; 100 contiguous axial slices; 1.5 mm isotropic voxel size; a 154 × 154 matrix size; b value = 1000 s/mm^2^; 60 diffusion encoding directions and a single baseline image acquired at 0 s/mm^2^. In addition, field map images were generated to correct the distortions caused by field inhomogeneities (TE 1/TE 2 = 4.92/7.38 ms, with the same slice prescription, slice thickness and field of view as the DWI sequence).

White matter (WM) lesions were segmented in the 3D-MPRAGE space with the JIM 7 software (http://www.xinapse.com/j-im-7-software/) using 3D-FLAIR images as a reference to improve the identification and delineation of the lesions. A WM lesion-filling approach^24^ was applied and for the network analysis, 76 structural brain labels were obtained using the Mindboogle (https://mindboggle.info/) and FSL-FIRST packages.

#### Advanced fibre tracking method to estimate the edges of the network

Pre-processing of diffusion MRI was achieved by DWI denoising, motion correction and geometrically unwrapping of the images^25^. Structural brain network reconstruction was performed using the multi-tissue constrained spherical deconvolution (MT-CSD) method to decompose two different tissue components, WM (anisotropic) and grey matter (GM)/cerebrospinal fluid (isotropic), which was followed by high-order probabilistic tractography using the MRtrix3 software package (http://www.mrtrix.org/)^25^. The WM and lesions mask were defined as tractography seeding^16^ to guarantee the streamline generation over pathological tissue. Using the anatomical constrained tractography (ACT) framework^26^, with the stopping criteria of a fibre orientation distribution (FOD) amplitude equal to 0.06, a set of 6 million streamlines was generated by the fibre tracking method. To minimise the number of false positives streamlines, we applied an anatomical exclusion criteria post-processing^27^.

#### Connectome matrix reconstruction

The parcellation scheme (76 regions) of structural images were adjusted to the native DWI space to define the nodes of the network. Structural brain networks were then represented by 76 × 76 weighted connectivity matrices based on the mean fractional anisotropy (FA) values for each connection (2850 network edges), obtained from lesional and normal appearing brain tissue. We incorporated a weighting factor for each streamline contribution^28^. Only connections present in more than 60% of the HV cohort were included in the statistical analyses^29^. Finally, age and gender correction was performed by regression analysis applied to each FA connectivity matrix.

#### Network analyses

Using iGraph (https://igraph.org/), we obtained five node-based common graph measures^30^ from each participant’s FA-weighted connectivity matrix computed for each of the 76 regions from the parcellation map: node strength, the sum of the weights of all edges connected to a node; local efficiency, the average inverse shortest path length or global efficiency of a node calculated from the subgraph created by the node’s neighbours; clustering coefficient, the fraction of the triangles present around a node; betweenness centrality, the fraction of all the shortest path in the graph that pass through a node; and node degree, the total number of edges connected to a node.

#### Classification using SVM with the radial basis function kernel

We defined two groups to build the classifiers, the first comprised of all MS patients relative to the HVs, and the second, to compare CI and CP patients. The construction of each SVM model implied three steps, the first of which involved the selection of the graph features, which aimed to reduce input dimensionality, particularly recommended when the number of features is higher than the number of samples and when most of the data are usually redundant or irrelevant^31^ and hence, to minimise the overlap between groups and achieve greater dissimilarity. We only selected those graph metrics for which group comparison returned significant differences (corrected p < 0.05) when comparing CP patients with HVs, or CI with CP patients^32^.

The second step consisted of instance creation and, given the imbalance in the number of subjects in each group, we built 100 instances of the SVM model. In order to achieve a balance, we performed random undersampling over the largest group. Thus, for the classification of MS patients relative to HVs, each instance included 90 random stratified observations (45 HV, 20 CI and 25 CP patients) and 168 observations for the classification of CI relative to CP patients (84 CI and 84 CP patients). To find the optimal input parameters for the SVM, namely C and γ, a grid search was performed using growing sequences.

The third step was validation using *k*-fold cross-validation (with *k* = *10*). Thus, at each iteration of the first type of classification, 81 observations were used for training and 9 for testing, while for the second type of classification, 152 observations were used for training and 16 for testing. We obtained several performance indicators for each validation test, including accuracy, sensitivity, specificity and F1-score^33^. These results were further analysed using other confusion matrix indicators to assess the overall performance of the classifiers.

#### Statistical analysis

The Shapiro-Wilks method was used to assess normality. Differences in demographic, clinical and neuroimaging data among the groups were studied using a chi-squared test, one-way ANOVA or Kruskal-Wallis tests, as appropriate, with a significance level set to p < 0.05. Levene’s test for equality of variances was used to assess the homoscedasticity assumption and we used a Welch correction to compare group differences when this assumption was violated. Multiple comparisons were analysed with a Tukey HSD or Dunn’s test as necessary, and all statistical analyses were performed using the R Statistical Software (www.R-project.org).

## Results

The demographic, clinical and cognitive data of the cohort were collected (Table 1) and of the MS patients, 104 (55%) were classified as CP and 84 (45%) as CI. As expected, the group of CI patients were older, more disabled and had a higher proportion of progressive MS patients (p < 0.05, Table 1).

**Table 1:** Demographic, clinical and cognitive data of the participants.

	Healthy volunteers (n = 45)	Cognitive preserved (n = 104)	Cognitive impaired (n = 84)	*p*-value
Age, years	37.77 ± 11.01	41.90 ± 9.07	44.57 ± 11.27	0.003^b^
Female, n (%)	27 (60)	77 (74)	52 (62)	0.116^a^
Type of MS, n (%): RRMS SPMS	—	99 (95) 5 (5)	71 (85) 13 (15)	0.026^a^
Disease duration, years	—	11.04 ± 9.03	13.97 ± 10.17	0.044^c^
Median EDSS score (range)	—	2.0 (0.0–6.5)	2.5 (0.0–6.5)	0.009^d^
Lesion volume (cm^3^)	—	6.26 ± 6.98	12.52 ± 15.00	<0.001^c^
Grey matter volume (cm^3^)	826.07 ± 54.56	794.38 ± 52.25	767.82 ± 68.48	<0.001^e^
Global cognition z-score	—	0.014 ± 0.436	−1.099 ± 0.571	<0.001^c^

Continuous variables are given as the mean ± standard deviation: EDSS = Expanded Disability Status Scale; RRMS = relapsing remitting multiple sclerosis; SPMS = secondary progressive multiple sclerosis. a, Chi square test; b, Student’s t-test for independent samples; c, Mann-Whitney U Test; d, Wilcoxon rank-sum test; e, One-way analysis of variance.

### Differences in node-based graph metrics among the groups

**Figure 1:**
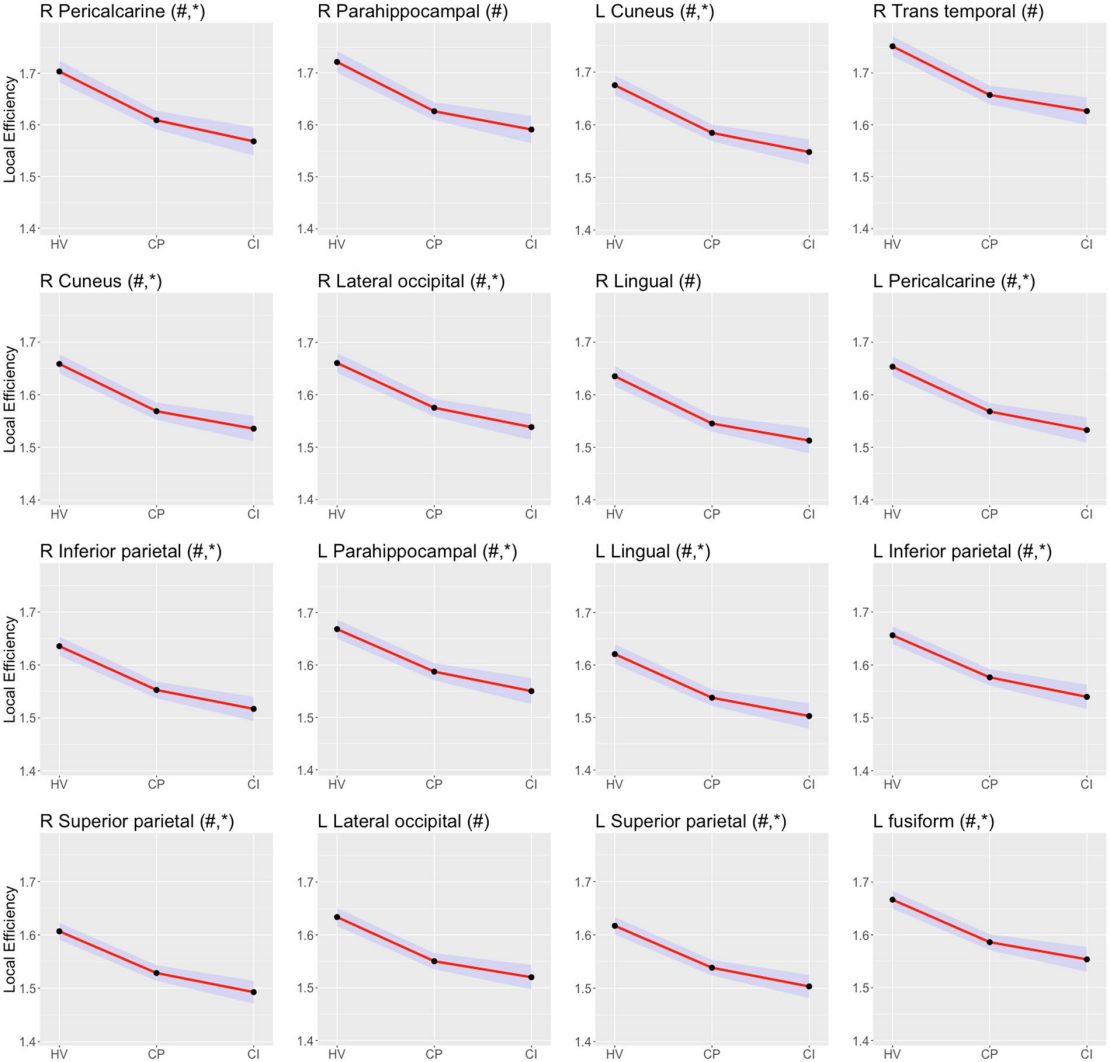
Mean and 95% confidence interval for the local efficiency in healthy volunteers (HV), cognitive preserved (CP) and cognitive impaired patients (CI) patients. For each node, ‘#’ stands for the statistical significance between CP and HV, and ‘*’ for CI vs. CP.

Most differences among the groups were observed in the measures of local efficiency and node strength (corrected p < 0.05). Local efficiency was reduced in CP patients relative to the HVs in all the nodes studied (corrected p < 0.05), while in CP compared to CI patients local efficiency was decreased in 42 (55.3%) of the studied nodes (corrected p < 0.05). Regions involving bilateral pericalcarine, cuneus and lateral occipital cortex, and right parahippocampal, lingual and transverse temporal cortex were the regions with the largest differences between CP patients and HVs (corrected p < 0.05). On the other hand, areas associated with the pericalcarine, and the superior and inferior parietal cortex of both hemispheres, the right lateral occipital cortex, and the left parahippocampal cortex and cuneus were those with the largest differences between the two MS groups (corrected p < 0.05, see Fig. 1 for the results from the nodes with largest differences between CI and CP patients).

**Figure 2:**
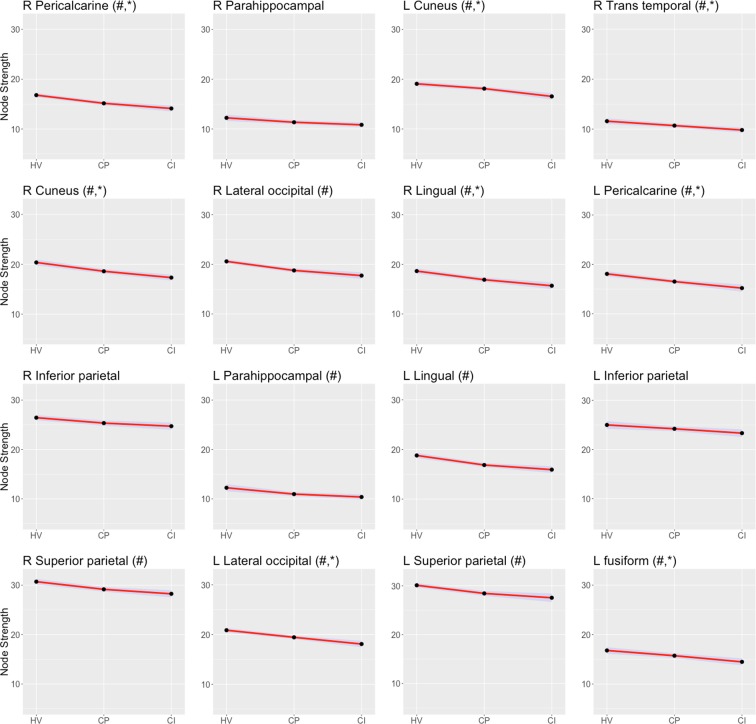
Mean and 95% confidence interval of node strength results from healthy volunteers (HV), cognitive preserved (CP) and cognitive impaired patients (CI) patients. For each node, ‘#’ stands for statistical significance between CP and HV, and ‘*’ for CI vs. CP.

In terms of node strength, 53 (69.7%) nodes were weaker in CP patients than in the HVs (corrected p < 0.05), and 44 (57.9%) nodes in CI patients were weaker than in CP patients (corrected p < 0.05). Areas such as the nucleus accumbens, hippocampus and lingual gyrus from both hemispheres, right cuneus and pericalcarine cortex and the left parahippocampal area were the regions with the largest differences between CP and HVs (corrected p < 0.05). Finally, the nodes in which there was the largest differences between MS groups were the bilateral hippocampus, right precuneus, occipital cortex (bilateral cuneus, right lingual, left pericalcarine and left lateral occipital cortex), the temporal cortex (right superior temporal and left fusiform) and the bilateral isthmus of the cingulate cortex (corrected p < 0.05, Fig. 2).

With the clustering coefficient, as well as for betweenness centrality and node degree, a limited number of nodes displayed significant differences (corrected p < 0.05). Thus, 12 (15.8%), 10 (13.2%) and 13 (17.1%) nodes were different in CP patients relative to HVs respectively, and 6 (7.9%), 8 (10.5%) and 10 (13.2%) between the CI and CP patients. Clustering coefficient and node degree were increased in CP compared to HV in 11 and 12 nodes, respectively, and between CI and CP patients in 6 and 10 regions. Moreover, 2 nodes showed higher betweenness centrality in CP compared to HV and 5 nodes in CI compared to CP patients (corrected p < 0.05, Supplementary Figs. 1–3).

### SVM classification

**Figure 3:**
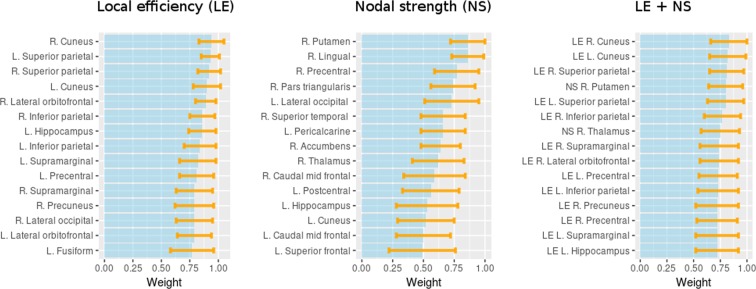
Bar plots of the 15 feature weights with the highest means, considering the 100 SVM models and comparing MS patients to HVs. The models are based on local efficiency (LE), node strength (NS), or both. The bars indicate the mean values while the orange lines show the ranges: mean ± standard deviation.

Using the SVM-based classification method to differentiate MS patients from HVs, and with the network measures with larger differences among the groups as inputs, a maximal discrimination accuracy of 77.15% was obtained for local efficiency and of 74.84% for node strength (corrected p < 0.05 when comparing CP with HV and CI with CP). The accuracy achieved in classifying CI and CP patients was 59.46% using local efficiency and 60.77% using node strength (see Table 2 for the accuracy, precision, sensitivity and specificity obtained from these models). When the SVM used the combined information of local efficiency and node strength, the accuracy to differentiate MS patients from HV was 76.88%, while it was 59.90% to differentiate CI from CP patients (Table 2). The features weights with the highest means for local efficiency, node strength and both properties together that were obtained with the different SVM executions are also shown (Fig. 3).

**Table 2:** Measures of the SVM classification performance based on measures of local efficiency (LE), node strength (NS), or both.

Groups	MS patients vs. HVs			CI vs. CP patients		
Measures	LE	NS	LE + NS	LE	NS	LE + NS
Accuracy	77.15 ± 3.35	74.84 ± 3.11	76.88 ± 3.35	59.46 ± 1.64	60.77 ± 1.44	59.90 ± 1.25
Sensitivity	74.27 ± 7.85	69.66 ± 7.28	72.17 ± 7.01	39.31 ± 13.19	46.07 ± 17.21	36.91 ± 13.40
Specificity	80.01 ± 3.77	79.94 ± 5.43	81.53 ± 3.85	79.62 ± 11.59	75.47 ± 16.31	82.89 ± 12.90
F1-score	75.99 ± 4.37	72.67 ± 4.27	74.90 ± 4.52	49.65 ± 9.12	52.29 ± 9.17	46.65 ± 8.31
Number of features	42	33	75	42	33	75

The results are presented as the mean percentage value ± standard deviation of the 100 instances with different subjects from the largest group using *k*-folding cross-validations: CI = cognitive impaired; CP = cognitive preserve; HV = healthy volunteers.

## Discussion

Using small-worldness and segregation measures, this study demonstrates changes in brain structural connectivity in a cohort of MS patients, even in the absence of cognitive decline. Patients with cognitive deficits experience more prominent modifications to their structural network, involving areas related to eloquence, suggesting a collapse of the network that drives cognitive dysfunction. Indeed, changes in these areas can discriminate MS patients from healthy individuals, although the capacity of these measures to differentiate patients based on their cognitive status is more restricted, suggesting that other factors may also influence such cognitive disturbances.

Local efficiency, a measure of how adjacent nodes are interconnected, appears to be a sensitive measure to study structural network changes during the disease course, with an increase in the early stages^13^ followed by a widespread reduction as the disease progresses, even when cognitive impairment is not yet established. Moreover, a reduction in node strength was also associated with disease duration in previous reports^13^. The findings of the present study support a general reduction in connectivity in MS patients that would appear to impair short-range connections between nearby regions. These changes are apparently larger than those identified earlier in individuals with a shorter disease duration^2^ and hence, reflect the vulnerability of the structural network to disease burden^3^.

Network modifications can be considered as a hallmark of the disease and they may serve to identify patients with MS, or even their cognitive status. When we applied SVM techniques to assess this possibility, we achieved a maximum accuracy of 76.8% based on local efficiency. Classification measures with different accuracies to distinguish MS patients from HVs by applying graph metrics from both structural and functional networks have been described previously^7^, as well as the efforts to distinguish clinically isolated syndrome from MS through the WM and GM connectivity^8^. The present results are in line of findings centred in diffusion-based structural connectivity, which reported an accuracy of 72.5% discriminating patients with MS from healthy individuals^7^, but also, they provide valuable information on the areas that were most distinct between the groups and thus, those that suffer characteristic damage in association with the disease. The power and the accuracy of the predictions from any machine learning-based algorithm are directly linked to the quality and size of the training sample^10,14^. In this sense, this study focused on a large sample of MS patients, which enhances the variance and reduces the bias that complicates the learning task associated with machine learning-based methods. SVM is a popular automatic classification method based on machine learning. Its simplicity favours its application to a wide range of discrimination tasks through the use of MRI data^7–9,12^. Using structural connectivity with advanced tractography techniques (MT-CSD), and no specific significance level limiting the values, the data demonstrated the viability of the approach adopted in a research environment, and the potential for its future translation into clinical practise as a complementary tool for subjects stratification.

Patients with cognitive impairment had a larger reduction in local efficiency and node strength in nodes that were already affected. Such modifications support the vulnerability of impaired regions to damage, and the hypothesis that when such damage is more intense, cognitive deficits arise due to the collapse of the network or its inability to compensate the brain impairment^6^. Reduced connectivity at nodes involving the parieto-occipital and medial temporal areas are the largest differences found in cognitively impaired MS patients. Most of these regions are considered brain hubs, maintaining a large number of connections across the brain and with intense metabolic demands, which increases their vulnerability to brain damage^15^. Hence, the loss of local integrity in these areas may be related to neuronal disorganization that drives cognitive impairment. The vulnerability of the frontal-parietal network components to neuroaxonal and demyelination damage was described in an earlier study we performed, along with its relationship to cognition in MS patients^2,16^.

However, the accuracy of machine learning techniques to distinguish patients based on their disability was more limited, as the mean accuracy was 51% (standard deviation 8.6) with *k*-folding cross-validations, and the maximal accuracy was 60.77% using nodal strength to classify CI and CP MS patients. The difficulty in discriminating patients with different cognitive status using only MRI diffusion characteristics demonstrates the moderate relationship between structural connectivity data and cognition and the low specificity of those changes, that partially overlap between groups. Moreover, this situation highlights the need to take into account other important parameters that may influence the relationship between brain damage and function, such as brain plasticity or cognitive reserve^17^.

One limitation of this study was that FA, the metric from which graph values were calculated is difficult to interpret and it has a limited capacity to estimate changes in the underlying microanatomy due to confounding factors, such as those derived from fibre crossing or orientation dispersion^18^. Recently, multi-shell DWI methods were shown to be capable of detecting potentially relevant network changes in early MS^19^. Further studies with these techniques may better characterise the brain microstructure in the context of this complex disease^20^. To improve the accuracy in classifying patients based on their cognitive status, future analyses may include clinical and other neuroimaging metrics from larger cohorts with stronger cognitive differences in a model that could modulate the changes in connectivity. However, this study focused on investigating the changes in network properties produced by the disease, relating them to cognition and their discriminative ability. Finally, the HV group sample size was quite small compared to the MS group, thus further studies with larger sample sizes and more advanced forms of the disease are advisable to validate our findings.

Our results showed a widespread reduction in the structural integrity of the brain network in patients with MS, even when cognitive decline is not yet established. Patients with impaired cognitive performance have worse brain connectivity, suggesting that as structural damage increases in vulnerable and eloquent regions, network collapse and cognitive deficits flourish. In addition, we demonstrate the feasibility of applying SVM methods based only on central structural network features to distinguish between HVs and MS patients. Future work should focus on improving the accuracy to distinguish the different cognitive profile of patients by adding more advanced microstructural features from diffusion MRI.

## Supplementary information

Supplementary information.

## References

[1] Li Y (2013). Diffusion tensor imaging based network analysis detects alterations of neuroconnectivity in patients with clinically early relapsing-remitting multiple sclerosis. Hum. Brain Mapp..

[2] Llufriu S (2017). Structural networks involved in attention and executive functions in multiple sclerosis. NeuroImage: Clinical.

[3] Charalambous T (2019). Structural network disruption markers explain disability in multiple sclerosis. J. Neurol. Neurosurg. Psychiatry.

[4] Pagani, E. *et al*. Structural connectivity in multiple sclerosis and modeling of disconnection. *Mult. Scler*. 1352458518820759 (2019). 10.1177/1352458518820759 30625050

[5] Fleischer V (2019). Graph Theoretical Framework of Brain Networks in Multiple Sclerosis: A Review of Concepts. Neuroscience.

[6] Schoonheim MM, Meijer KA, Geurts JJG (2015). Network collapse and cognitive impairment in multiple sclerosis. Front. Neurol..

[7] Zurita M (2018). Characterization of relapsing-remitting multiple sclerosis patients using support vector machine classifications of functional and diffusion MRI data. Neuroimage Clin.

[8] Muthuraman *et al*. Structural Brain Network Characteristics Can Differentiate CIS from Early RRMS. *Frontiers in Neuroscience* **10** (2016). 10.3389/fnins.2016.00014 PMC4735423 26869873

[9] Kocevar, G. *et al*. Graph Theory-Based Brain Connectivity for Automatic Classification of Multiple Sclerosis Clinical Courses. *Frontiers in Neuroscience* **10** (2016). 10.3389/fnins.2016.00478 PMC5078266 27826224

[10] Vapnik, V. N. *Statistical learning theory*. (Wiley-Interscience, 1998).

[11] Bendfeldt Kerstin, Taschler Bernd, Gaetano Laura, Madoerin Philip, Kuster Pascal, Mueller-Lenke Nicole, Amann Michael, Vrenken Hugo, Wottschel Viktor, Barkhof Frederik, Borgwardt Stefan, Klöppel Stefan, Wicklein Eva-Maria, Kappos Ludwig, Edan Gilles, Freedman Mark S., Montalbán Xavier, Hartung Hans-Peter, Pohl Christoph, Sandbrink Rupert, Sprenger Till, Radue Ernst-Wilhelm, Wuerfel Jens, Nichols Thomas E. (2018). MRI-based prediction of conversion from clinically isolated syndrome to clinically definite multiple sclerosis using SVM and lesion geometry. Brain Imaging and Behavior.

[12] Wottschel V (2015). Predicting outcome in clinically isolated syndrome using machine learning. Neuroimage Clin.

[13] Fleischer V (2017). Increased structural white and grey matter network connectivity compensates for functional decline in early multiple sclerosis. Mult. Scler..

[14] Wu, G., Shen, D. & Sabuncu, M. *Machine Learning and Medical Imaging*. (Academic Press, 2016).

[15] Crossley NA (2014). The hubs of the human connectome are generally implicated in the anatomy of brain disorders. Brain.

[16] Solana E (2018). Magnetic resonance markers of tissue damage related to connectivity disruption in multiple sclerosis. Neuroimage Clin.

[17] Mollison D (2017). The clinico-radiological paradox of cognitive function and MRI burden of white matter lesions in people with multiple sclerosis: A systematic review and meta-analysis. PLoS One.

[18] Wheeler-Kingshott CAM, Cercignani M (2009). About ‘axial’ and ‘radial’ diffusivities. Magn. Reson. Med..

[19] Tur, C. *et al*. A multi-shell multi-tissue diffusion study of brain connectivity in early multiple sclerosis. *Mult. Scler*. 1352458519845105 (2019). 10.1177/1352458519845105 PMC7611366 31074686

[20] Alexander DC, Dyrby TB, Nilsson M, Zhang H (2019). Imaging brain microstructure with diffusion MRI: practicality and applications. NMR Biomed..

[21] Polman CH (2011). Diagnostic criteria for multiple sclerosis: 2010 revisions to the McDonald criteria. Ann. Neurol..

[22] Rao SM, Leo GJ, Bernardin L, Unverzagt F (1991). Cognitive dysfunction in multiple sclerosis. I. Frequency, patterns, and prediction. Neurology.

[23] Sepulcre J (2006). Cognitive impairment in patients with multiple sclerosis using the Brief Repeatable Battery-Neuropsychology test. Mult. Scler..

[24] Battaglini M, Jenkinson M, De Stefano N (2012). Evaluating and reducing the impact of white matter lesions on brain volume measurements. Hum. Brain Mapp..

[25] Tournier, J.-D. *et al*. MRtrix3: A fast, flexible and open software framework for medical image processing and visualisation, 10.1101/551739 10.1016/j.neuroimage.2019.116137 31473352

[26] Smith RE, Tournier J-D, Calamante F, Connelly A (2012). Anatomically-constrained tractography: improved diffusion MRI streamlines tractography through effective use of anatomical information. Neuroimage.

[27] Martínez-Heras E (2015). Improved Framework for Tractography Reconstruction of the Optic Radiation. PLoS One.

[28] Smith RE, Tournier J-D, Calamante F, Connelly A (2015). SIFT2: Enabling dense quantitative assessment of brain white matter connectivity using streamlines tractography. Neuroimage.

[29] de Reus MA, van den Heuvel MP (2013). Estimating false positives and negatives in brain networks. Neuroimage.

[30] Mijalkov M (2017). BRAPH: A graph theory software for the analysis of brain connectivity. PLoS One.

[31] Fodor, I. K. A Survey of Dimension Reduction Techniques., 10.2172/15002155 (2002).

[32] Hastie, T., Tibshirani, R. & Friedman, J. *The Elements of Statistical Learning: Data Mining, Inference, and Prediction, Second Edition*. (Springer Science & Business Media, 2009).

[33] Van Rijsbergen, C. J. *Information Retrieval*. (Butterworth-Heinemann, 1979).

