# Supplementary material for: Modified connectivity of vulnerable brain nodes in multiple sclerosis, their impact on cognition and their discriminative value
Source: Sci Rep. 2019 Dec 27;9:20172. doi: 10.1038/s41598-019-56806-z (PMC6934774; doi:10.1038/s41598-019-56806-z)
Supplement: Supplementary file 1 — Supplementary information. [file 41598_2019_56806_MOESM1_ESM.pdf]

Modified connectivity of vulnerable brain nodes in multiple sclerosis, their impact on cognition and their discriminative value.

Elisabeth Solana[1a], Eloy Martinez-Heras[1a], Jordi Casas-Roma[2], Laura Calvet[2], Elisabet Lopez-Soley[1], Maria Sepulveda[1], Nuria Sola-Valls[1], Carmen Montejo[1], Yolanda Blanco[1], Irene Pulido-Valdeolivas[1], Magi Andorra[1], Albert Saiz[1], Ferran Prados[2,3,4], Sara Llufriu[1]*.

1. Center of Neuroimmunology, Laboratory of Advanced Imaging in Neuroimmunological Diseases, Hospital Clinic Barcelona, Institut d'Investigacions Biomèdiques August Pi i Sunyer (IDIBAPS) and Universitat de Barcelona. Barcelona. Spain

2. E-health Centre, Universitat Oberta de Catalunya. Barcelona. Spain.

3. Centre for Medical Image Computing (CMIC), Department of Medical Physics and Bioengineering, University College London, London, UK.

4. NMR Research Unit, Queen Square MS Centre, Department of Neuroinflammation, UCL Institute of Neurology, University College London, London, UK.

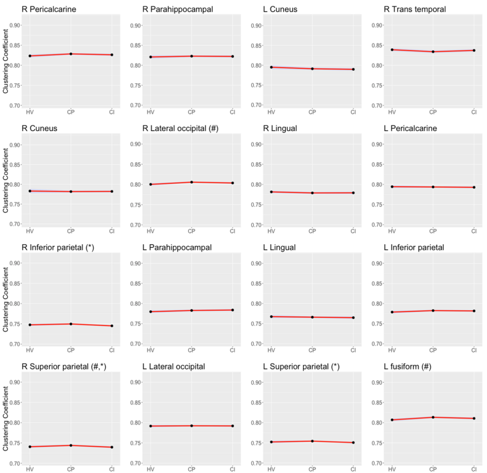
R Pericalcarine
R Parahippocampal
L Cuneus
R Trans temporal
R Cuneus
R Lateral occipital (#)
R Lingual
L Pericalcarine
R Inferior parietal (*)
L Parahippocampal
L Lingual
L Inferior parietal
R Superior parietal (#,*)
L Lateral occipital
L Superior parietal (*)
L fusiform (#)
Clustering Coefficient
0.90
0.85
0.80
0.75
0.70
HV
CP
CI

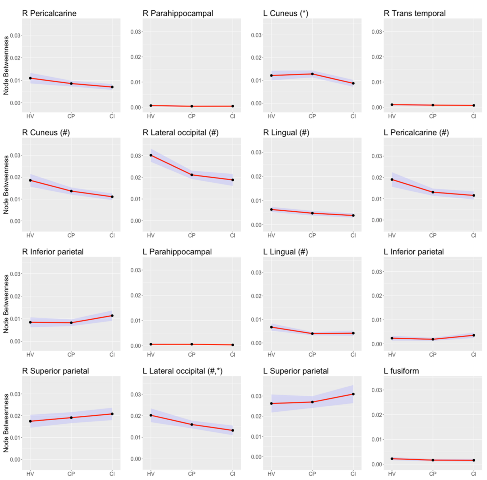

R Pericalcarine
R Parahippocampal
L Cuneus (*)
R Trans temporal
R Cuneus (#)
R Lateral occipital (#)
R Lingual (#)
L Pericalcarine (#)
R Inferior parietal
L Parahippocampal
L Lingual (#)
L Inferior parietal
R Superior parietal
L Lateral occipital (#,*)
L Superior parietal
L fusiform
Node Betweenness
0.00
0.01
0.02
0.03
HV
CP
CI

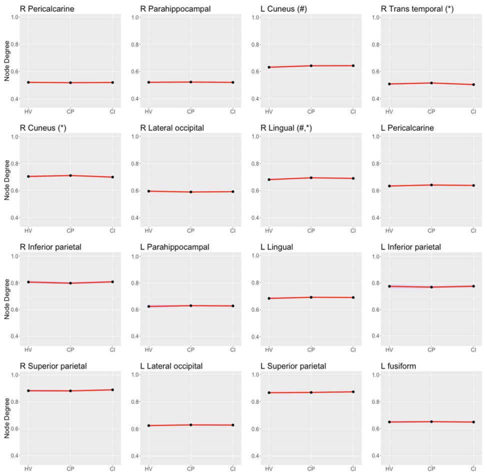
R Pericalcarine
R Parahippocampal
L Cuneus (#)
R Trans temporal (*)
R Cuneus (*)
R Lateral occipital
R Lingual (#,*)
L Pericalcarine
R Inferior parietal
L Parahippocampal
L Lingual
L Inferior parietal
R Superior parietal
L Lateral occipital
L Superior parietal
L fusiform
Node Degree
HV
CP
CI
0.4
0.6
0.8
1.0
